# Effect of *MTTP* -493G/T, I128T, Q95H and Q244E polymorphisms on hepatic steatosis in patients with chronic hepatitis

**DOI:** 10.1016/j.clinsp.2022.100094

**Published:** 2022-08-23

**Authors:** Thamiris Vaz Gago Prata, Caroline Manchiero, Bianca Peixoto Dantas, Arielle Karen da Silva Nunes, Fátima Mitiko Tengan, Mariana Cavalheiro Magri

**Affiliations:** a Laboratorio de Investigacao Médica em Hepatologia por Virus (LIM-47), Hospital das Clínicas, Faculdade de Medicina, Universidade de São Paulo (HCFMUSP), São Paulo, SP, Brazil; b Departamento de Molestias Infecciosas e Parasitarias, Faculdade de Medicina, Universidade de São Paulo (FMUSP), São Paulo, SP, Brazil

**Keywords:** Chronic hepatitisC, Genetic models of inheritance, Hepatic steatosis, *Microsomal Triglyceride Transfer Protein* (*MTTP*), Single Nucleotide Polymorphisms (SNPs)

## Abstract

**Background:**

Chronic hepatitis C is characterized by a progressive deterioration of liver function and is involved in metabolic complications, such as hepatic steatosis.

**Objective:**

The aim of this study was to investigate the role of host and viral characteristics associated with -493G/T (rs1800591), I128T (rs3816873), Q95H (rs61733139), and Q244E (rs17599091) Single Nucleotide Polymorphisms (SNPs) in the *Microsomal Triglyceride Transfer Protein* (*MTTP*) gene on hepatic steatosis in chronic hepatitis C.

**Methods:**

SNPs were genotyped by PCR-RFLP and analyzed in combination with host and viral characteristics by multiple logistic regression in different genetic models of inheritance.

**Results:**

The authors analyzed 236 patients with chronic hepatitis C, and 53% had hepatic steatosis. The mutated allele frequencies were > 5%, and the genotypes were in Hardy-Weinberg equilibrium (p ≥ 0.05). It was observed that patients with HCV genotype 3 infection (OR = 2.74, 95% CI 1.24‒6.06, *p* = 0.013), female sex (OR = 2.28, 95% CI 1.21‒4.28, *p* = 0.011) and moderate- and high-intensity liver inflammatory activity (A2-A3) (OR = 3.61, 95% CI 1.86‒7.01, *p* < 0.001) alone exhibited a higher risk of steatosis. The results of multiple logistic regression analysis for interaction showed that for the -493G/T SNP, when the GT/TT genotype (dominant model) and the GT genotype (codominant model) were each combined with HCV genotype 3 infection, an 11.51-fold (95% CI 2.08‒63.59, *p* = 0.005) and a 15.69-fold (95% CI 2.46‒99.85, *p* = 0.004) increased risk of steatosis, respectively, was observed. For the I128T SNP, when both the IT/TT genotype (dominant model) and the IT genotype (codominant model) were combined with HCV genotype 3 infection, an 8.51-fold (95% CI 1.59‒45.54, *p* = 0.012) and an 8.40 fold (95% CI 1.51‒46.91, *p* = 0.015) increased risk of steatosis, respectively, was observed.

**Conclusion:**

The present study showed that the viral genotype combined with the -493G/T and I128T SNPs in the *MTTP* gene influences hepatic steatosis.

## Highlights

• Important etiologies of chronic liver disease are viral hepatitis.
• Viral hepatitis B and C causes 1.1 million deaths per year.
• Hepatic steatosis (liver fat accumulation) is a metabolic complication of hepatitis C.
• The underlying mechanisms involving steatosis include genetic polymorphisms.
• -493G/T and I128T polymorphisms in the *MTTP* gene are relevant in hepatic steatosis.

## Introduction

Chronic liver disease is characterized by a progressive deterioration of liver function for more than six months, and this process is related to the persistent inflammation, destruction, and regeneration of the liver parenchyma. The most common etiologies are the presence of alcoholic liver disease, autoimmune or genetic causes, drugs, Nonalcoholic Fatty Liver Disease (NAFDL)/Nonalcoholic Steatohepatitis (NASH), and chronic viral hepatitis.^1^ Regarding viral hepatitis, hepatitis C causes inflammation in the liver that is caused by Hepatitis C Virus (HCV). According to the World Health Organization (WHO), an estimated 58 million people are chronically infected with HCV worldwide.^2^ Viral hepatitis (hepatitis B and C) causes 1.1 million deaths per year and 3.0 million new infections. Among hepatitis C patients, approximately 62% of those diagnosed receive specific treatment.^3^ In addition, hepatitis C is involved in several metabolic complications, such as insulin resistance, hepatic steatosis, hyperlipidemia, and metabolic syndrome.^4^

The development of hepatic steatosis influences disease progression. In addition, persistence and even an increase in the degree of steatosis are observed in patients who achieve a Sustained Virologic Response (SVR) after specific treatment with Direct-Acting Antiviral (DAA) drugs one year after treatment ends.^5^, ^6^, ^7^ Interestingly, diabetes mellitus was reported to be a factor that independently affected liver stiffness after DAA treatment despite SVR.^8^ The underlying mechanisms involving the presence of steatosis, especially before treatment, can be alcohol consumption, being overweight, obesity, diabetes type 2 mellitus, HCV genotype, Human Immunodeficiency Virus (HIV) coinfection, and genetic polymorphisms in genes, such as *Transmembrane six Superfamily member* 2 (*TM6SF2*), *Patatin-Like Phospholipase Domain Containing* 3 (*PNPLA3*) and *Microsomal Triglyceride Transfer Protein* (*MTTP*).^9^, ^10^, ^11^, ^12^, ^13^ In this context, the role of host and viral characteristics associated with genetic polymorphisms in liver fat accumulation in chronic hepatitis C was investigated in the present study. The aim of the present study was to determine whether the -493G/T (rs1800591), I128T (rs3816873), Q95H (rs61733139), and Q244E (rs17599091) Single Nucleotide Polymorphisms (SNPs) in the *MTTP* gene are linked to the presence of hepatic steatosis in patients with chronic hepatitis C.

## Materials and methods

### Patient selection

Patients with chronic hepatitis C were selected from the Clinical Hospital of the School of Medicine at the University of Sao Paulo (HCFMUSP) in Brazil. The present study was approved by the Ethics Committee (Ethics Committee for Analysis of Research Projects) of HCFMUSP. The protocol followed the guidelines of the 1975 Declaration of Helsinki, and informed consent was obtained from all participants. The actual research did not conflict with any treatment or medical advice. The present study is part of a sequence of previously published studies.^11^^,^^14^

The inclusion criteria were patients who presented positive anti-HCV antibody and HCV-RNA results for more than six months had undergone histopathological analysis after liver biopsy, and were older than 18 years. The exclusion criteria were patients who were coinfected with HIV or Hepatitis B Virus (HBV), patients who received previous HCV treatment, and the presence of liver conditions of other etiologies, such as autoimmune liver disease, primary biliary cirrhosis, and Wilson's disease. The patients were included in the study and had biological samples collected before initiating any hepatitis C treatment.

A total of 236 patients with chronic hepatitis C infection under follow-up at the outpatient clinic of infectious diseases in the HCFMUSP who met the criteria described were included from 2010 to 2012. The calculation of the minimum sample size required was performed considering the following parameters: the alpha value of 5%; a beta value of 20% and, consequently, a power of 80% and standard error type 2. The recommended equation for this type of study was used and was as follows: n = π (1-π)/e2.^15^ This calculation was performed considering the frequency of 27.5% of the recessive allele (T) for the -493G⁄T SNP in the *MTTP* gene as described by Mirandola et al.^16^ The minimum sample size was calculated to be 225 patients. At the time of enrollment, all patients with chronic hepatitis C attending this outpatient clinic underwent liver biopsy. After histopathological analysis of the liver fragment, the patients were divided into two groups as follows: patients with hepatic steatosis (n = 125) and patients without hepatic steatosis (n = 111).

### Data collection

Data regarding epidemiological and demographical factors and laboratory tests were collected from medical records. The reference values of biochemical tests were as follows: Alanine Aminotransferase (ALT) levels: ≥ 41 U/L, Aspartate Aminotransferase (AST) levels: ≥ 37 U/L, Gamma Glutamyl Transpeptidase (GGT) levels: ≥ 61 U/L, insulin levels: ≥ 25 µU/mL, glucose levels: >99 mg/dL, triglyceride levels: ≥ 200 mg/dL, total cholesterol levels: ≥ 200 mg/dL, High-Density Lipoprotein (HDL) levels: ≤ 60 mg/dL, Low-Density Lipoprotein (LDL) levels: ≥ 130 mg/dL, and Very-Low-Density Lipoprotein (VLDL) levels: ≥ 40 mg/dL. HOMA-IR index (homeostasis model assessment of insulin resistance) was calculated as fasting insulin levels (μU/mL) × fasting glucose levels (mmoL/L)/22.5, and its reference value was ≥ 3,0. The reference value for HCV viral load was <850,000 IU/mL, and that for alcohol consumption was ≥20 g/day. Metabolic syndrome was defined by the appearance of three or more of the following alterations: high triglyceride levels (≥ 150 mg/dL), low HDL levels (≤ 40 mg/dL), diagnosis of diabetes or fasting blood glucose levels ≥ 100 mg/dL and the diagnosis of high blood pressure or systolic blood pressure ≥ 130 mmHg and/or diastolic blood pressure ≥ 85 mmHg.

Histopathological analysis after liver biopsy of all patients was performed according to Kleiner et al.^17^ classification for the assessment of hepatic steatosis (graded as 0 to 3), according to the METAVIR^18^ classification for the assessment of hepatic fibrosis (graded as F0 to F4) and hepatic inflammatory activity (graded as A0 to A4), and according to Perls’ staining criteria (graded as 0 to 4) for the assessment of hepatic siderosis.

### Single nucleotide polymorphism genotyping

Peripheral blood (10 mL) was collected from patients with chronic hepatitis C from 2010 to 2012 and was stored at -80°C until processing. To genotype the -493G/T (rs1800591), I128T (rs3816873), Q95H (rs61733139) and Q244E (rs17599091) *MTTP* SNPs, Polymerase Chain Reaction-Restriction Fragment Length Polymorphism (PCR-RFLP) was performed. First, DNA was isolated using the ReliaPrep Blood gDNA Miniprep System according to the manufacturer's instructions (Promega, USA). The primers used during PCR to amplify the *MTTP* gene fragments have been described by Karpe et al.^19^ and Ledmyr et al.^20^ PCR was performed in a 16.7 μL reaction mix that included 5.3 μL of ultrapure water, 8.3 μL of GoTaq Green Master Mix (Promega, USA), 1.7 μL (30‒50 ng/μL) of isolated DNA and 0.7 μL (7 pmoL) of each primer. The number of PCR cycles was 40, and the annealing temperature varied between 53° and 60°C, according to the primer pair size and composition. PCR amplification was confirmed under Ultraviolet (UV) light by 3% agarose gel electrophoresis, and a 50 bp DNA molecular weight marker was utilized to validate the amplicon size.

The amplified PCR product was digested with a specific endonuclease restriction enzyme that recognizes one of the SNP alleles to cleave the amplified fragment at a specific site for subsequent differentiation of the alleles by size. The RFLP assay was performed in a 20 μL reaction mix containing 15 µL of PCR product, 2 µL of ultrapure water, 2 µL of the corresponding buffer of each enzyme, and 0.5‒1 µL of restriction enzyme according to the concentration used. The temperature and incubation time varied depending on the enzyme and the manufacturer's instructions. The restriction enzymes that were utilized have been previously described by Karpe et al.^19^ and Ledmyr et al.^20^ Enzymatic digestion was confirmed under UV light by 3% agarose gel electrophoresis. The genotypes expected for each *MTTP* SNP were as follows: -493G/T SNP, GG: 89 bp and 20 bp, GT: 109 bp, 89 bp, and 20 bp, TT: 109 bp; I128T SNP, II: 167 bp, IT: 167 bp, 138 bp, and 29 bp, TT: 138 bp and 29 bp; Q95H SNP, QQ: 148 bp and 35 bp, QH: 183 bp, 148 bp, and 35 bp, HH: 183 bp; Q244E SNP, QQ: 201 bp, QE: 201 bp, 149 bp, and 52 bp, EE: 149 bp and 52 bp. Quality control was used to verify the reproducibility of the results.

### Statistical analysis

For statistical analysis, IBM-SPSS version 20 software (IBM Corp., USA) and Hosmer; Lemeshow^21^ were used. The analysis of the Hardy-Weinberg equilibrium was performed using the Chi-Square test (p ≥ 0.05). All variables were categorized, and their frequencies were described. Bivariate and multiple logistic regression was used to determine the individual variables that could influence the presence of steatosis in patients with chronic hepatitis C. The Odds Ratio (OR) of each variable with the presence of hepatic steatosis was estimated with the respective 95% Confidence Interval (95% CI). The associations were evaluated in three different genetic models (codominant, dominant and recessive models) because the optimal genetic model of inheritance in genes in complex diseases has not been well established.

A bivariate and a multiple logistic regression model were used to determine which host and viral characteristics combined with each SNP (-493G/T, I128T, Q95H and Q244E) to influence the presence of steatosis in patients with chronic hepatitis C according to different genetic models. All variables independently associated with hepatic steatosis (*p* < 0.05) in multiple logistic regression and interactions that presented a significance level of 0.20 (*p* < 0.2) in the bivariate tests were included in the final multiple logistic regression model. In the multiple logistic regression model, the significance level was 0.05 (*p* < 0.05).

## Results

The study group consisted of 236 patients with chronic hepatitis C infection, 56.4% of whom were females, and the most frequent infection was by genotype non 3 (81.8%). Additionally, the most frequent self-reported ethnicity was white (81.0%). The distribution of patients with chronic hepatitis C considering the Body Mass Index (BMI) classification was as follows: 39.8% of the patients presented BMI < 25 kg/m^2^, which is considered a normal BMI and 60.2% of the patients presented BMI ≥ 25 kg/m^2^, which can be classified as overweight or obese. A total of 57.6% of patients exhibited elevated levels of ALT, 51.3% exhibited elevated levels of AST, and 44.1%. exhibited elevated levels of GGT. According to liver histology findings, 53% of the patients exhibited hepatic steatosis, 20.8% exhibited hepatic fibrosis stages of F3 and F4 and 66.1% exhibited hepatic inflammatory activity grades of A2 and A3 (Table 1).

**Table 1:** General characteristics of patients with chronic hepatitis C included in the study (total) and split according to the presence of hepatic steatosis, *n* (%).

Characteristic	Hepatic steatosis		Total
	No, n (%)	Yes, n (%)	n (%)
**n**	111 (47.0)	125 (53.0)	236 (100)
**Sex**			
Male	56 (54.4)	47 (45.6)	103 (43.6)
Female	55 (41.4)	78 (58.6)	133 (56.4)
**Age**			
< 50 years	40 (58.8)	28 (41.2)	68 (28.8)
≥ 50 years	71 (42.3)	97 (57.7)	168 (71.2)
**BMI**			
< 25 kg/m^2^	45 (48.9)	47 (51.1)	92 (39.8)
≥ 25 kg/m^2^	64 (46.0)	75 (54.0)	139 (60.2)
**Ethnicity**			
White	85 (44.5)	106 (55.5)	191 (81.2)
No White	25 (56.8)	19 (43.2)	44 (18.8)
**HOMA-IR**			
< 3	80 (52.6)	72 (47.4)	152 (65.5)
≥ 3	30 (37.5)	50 (62.5)	80 (34.5)
**Alcohol consumption**			
< 20 g/day	73 (49.3)	75 (50.7)	148 (62.7)
≥ 20 g/day	38 (43.1)	50 (56.9)	88 (37.3)
**Hypertension**			
No	76 (50.7)	74 (49.3)	150 (63.6)
Yes	35 (40.7)	51 (59.3)	86 (36.4)
**Diabetes mellitus**			
No	95 (47.5)	105 (52.5)	200 (84.7)
Yes	16 (44.4)	20 (55.6)	36 (15.3)
**HCV genotype 3**			
No	99 (51.3)	94 (48.7)	193 (81.8)
Yes	12 (27.9)	31 (72.1)	43 (18.2)
**HCV viral load**			
< 850,000 IU/mL	42 (45.2)	52 (54.2)	94 (41.2)
≥ 850,000 IU/mL	64 (47.8)	70 (52.2)	134 (58.8)
**ALT**			
< 41 U/L	54 (54.0)	46 (46.0)	100 (42.4)
≥ 41 U/L	57 (41.9)	79 (58.1)	136 (57.6)
**AST**			
< 37 U/L	67 (58.3)	48 (41.7)	115 (48.7)
≥ 37 U/L	44 (36.4)	77 (63.6)	121 (51.3)
**GGT**			
8‒61 U/L	70 (53.0)	62 (47.0)	132 (55.9)
> 61 U/L	41 (39.4)	63 (60.6)	104 (44.1)
**Total cholesterol**			
< 200 mg/dL	84 (46.7)	96 (53.3)	180 (76.3)
≥ 200 mg/dL	27 (48.2)	29 (51.8)	56 (23.7)
**LDL**			
< 130 mg/dL	91 (46.0)	107 (54.0)	198 (83.9)
≥ 130 mg/dL	20 (52.6)	18 (47.4)	38 (16.1)
**HDL**			
> 60 mg/dL	33 (44.0)	42 (56.0)	75 (31.8)
≤ 60 mg/dL	78 (48.4)	83 (51.6)	161 (68.2)
**VLDL**			
< 40 mg/dL	104 (47.3)	116 (52.7)	220 (93.2)
≥ 40 mg/dL	7 (43.8)	9 (56.3)	16 (6.8)
**Triglyceride**			
< 200 mg/dL	104 (47.1)	117 (52.9)	221 (93.6)
≥ 200 mg/dL	7 (46.7)	8 (53.3)	15 (6.4)
**Hepatic fibrosis**			
F0‒F2	97 (51.9)	90 (48.1)	187 (79.2)
F3‒F4	14 (28.6)	35 (71.4)	49 (20.8)
**Hepatic inflammatory activity**			
A0‒A1	57 (71.3)	23 (28.8)	80 (33.9)
A2‒A3	54 (34.6)	102 (65.4)	156 (66.1)
**Hepatic siderosis**			
No	106 (48.0)	115 (52.0)	221 (93.6)
Yes	5 (33.3)	10 (66.7)	15 (6.4)
**Metabolic syndrome**			
No	111 (47.5%)	123 (52.5%)	234 (99.1%)
Yes	0 (0.0%)	2 (100.0%)	2 (0.9%)

ALT, Alanine Aminotransferase; AST, Aspartate Aminotransferase; BMI, Body Mass Index; CI, Confidence Interval; GGT, Gamma Glutamyl Transpeptidase; HCV, Hepatitis C Virus; HDL, High-Density Lipoprotein; HOMA-IR, Homeostasis Model Assessment of Insulin Resistance; LDL, Low-Density Lipoprotein; OR, Odds Ratio; VLDL, Very Low-Density Lipoprotein.

The four SNPs (-493G/T, I128T, Q95H, and Q244E) evaluated in the *MTTP* gene in all patients were genotyped. For the -493G/T SNP, 109 (46.2%) patients exhibited the GG genotype (wild-type homozygous), 102 (43.2%) patients exhibited the GT genotype (heterozygous) and 25 (10.6%) patients exhibited the TT genotype (mutated homozygous). For the I128T SNP, 117 (49.6%) patients exhibited the II genotype (wild-type homozygous), 97 (41.1%) patients exhibited the IT genotype (heterozygous) and 22 (9.3%) patients exhibited the TT genotype (mutated homozygous). For the Q95H SNP, 196 (83.1%) patients exhibited the QQ genotype (wild-type homozygous), 39 (16.5%) patients exhibited the QH genotype (heterozygous), and 1 (0.4%) patient exhibited the HH genotype (mutated homozygous). For the Q244E SNP, 209 (88.6%) patients exhibited the QQ genotype (wild-type homozygous), and 27 (11.4%) patients exhibited the QE genotype (heterozygous); the EE genotype (mutated homozygous) was not detected in any participant included in the present study. Therefore, the frequency of mutated alleles of each SNP was > 5%, and the genotypes were distributed according to Hardy-Weinberg equilibrium (-493G/T: *p* = 0.875, I128T: *p* = 0.770, Q95H: *p* = 0.521 and Q95H: *p* = 0.351).

Bivariate and multivariate analyses were performed to individually assess which characteristics were independently associated with hepatic steatosis in patients with chronic hepatitis C (Supplementary file ‒ Tables S1 and S2). The multivariate analysis indicated that HCV genotype 3 infection (OR = 2.74, 95% CI 1.24‒6.06, *p* = 0.013) and female sex (OR = 2.28, 95% CI 1.21‒4.28, *p* = 0.011) were associated with a higher risk of hepatic steatosis. Additionally, patients with moderate- and high-intensity liver inflammatory activity (A2 and A3) exhibited a higher risk of hepatic steatosis than patients without or with low-intensity inflammatory activity (A0 and A1) (OR = 3.61, 95% CI 1.86‒7.01, *p* < 0.001). The variables that had a significant p-value were included in the interaction analysis (HCV genotype 3, female sex, and inflammatory activity A2-A3). In addition to these variables, HOMA-IR was included in the bivariate and multivariate analyses to assess the association of SNPs in the *MTTP* gene combined with different characteristics in the presence of hepatic steatosis (interaction analysis) due to its relevance to steatosis in chronic hepatitis C.

The results of the bivariate analysis of the characteristics combined with SNPs in the *MTTP* gene are presented in Table 2, Table 3, Table 4, Table 5. The characteristics combined with SNPs that had a significant statistical association with the presence of hepatic steatosis in this analysis were also included in the multiple logistic regression analysis for the *MTTP* -493G/T, I128T, Q95H and Q244E SNPs, and the results are described in Table 6, Table 7, Table 8, Table 9. Therefore, all variables associated alone with hepatic steatosis (p<0.05) in multiple logistic regression (Tables S2) and variables combined with SNPs (Table 2, Table 3, Table 4, Table 5) presented a significance level of 0.20 (*p* < 0.2) in the bivariate tests were included in the final multiple logistic regression model (interaction analysis) according to different genetic models (dominant, codominant and recessive models).

**Table 2:** Result of bivariate tests for interactions of the -493G/T SNP in the *MTTP* gene in different genetic models with characteristics of interest that influence the presence of hepatic steatosis in patients with chronic hepatitis C.

Parameter	OR	95% CI	p^a^
**Dominant model**			
-493G/T SNP GT/TT	1.05	0.63‒1.76	0.848
-493G/T SNP × Sex (female)	0,74	0.26‒2.09	0.562
-493G/T SNP × Age (≥ 50 years)	1.16	0.36‒3.78	0.803
-493G/T SNP × BMI (≥ 25 kg/m^2^)	1.05	0.36‒3.04	0.935
-493G/T SNP × HOMA-IR (≥ 3)	0.87	0.28‒2.64	0.800
-493G/T SNP × Hypertension	1.41	0.47‒4.17	0.539
-493G/T SNP × Diabetes mellitus	0.78	0.17‒3.54	0.751
-493G/T SNP × HCV genotype 3	9.74	2.02‒46.90	**0.005**
-493G/T SNP × ALT (≥ 41 U/L)	1.38	0.49‒3.92	0.544
-493G/T SNP × AST (≥ 37 U/L)	1.86	0.65‒5.33	0.249
-493G/T SNP × GGT (≥ 61 U/L)	1.93	0.68‒5.50	0.219
-493G/T SNP × Total cholesterol (≥ 200 mg/dL)	0.36	0.11‒1.23	0.103
-493G/T SNP × LDL (≥ 130 mg/dL)	0.45	0.11‒1.85	0.267
-493G/T SNP × HDL (≤ 60 mg/dL)	0.67	0.22‒2.02	0.472
-493G/T SNP × VLDL (≥ 40 mg/dL)	2.71	0.33‒22.30	0.354
-493G/T SNP × Triglyceride (≥ 200 mg/dL)	2.22	0.26‒18.85	0.464
-493G/T SNP × Fibrosis (F3‒F4)	2.22	0.56‒8.84	0.260
-493G/T SNP × Inflammatory activity (A2‒A3)	2.73	0.83‒8.97	0.098
-493G/T SNP × Siderosis	1.44	0.15‒13.53	0.750
**Codominant model**			
-493G/T SNP GG × GT	0.95	0.55‒1.63	0.849
-493G/T SNP GG × TT	1.62	0.66‒3.99	0.292
-493G/T SNP (GG × GT) × Sex (female)	0.74	0.25‒2.22	0.589
-493G/T SNP (GG × TT) × Sex (female)	0.67	0.11‒4.16	0.665
-493G/T SNP (GG × GT) × Age (≥ 50 years)	1.15	0.33‒4.01	0.824
-493G/T SNP (GG × TT) × Age (≥ 50 years)	1.08	0.11‒10.70	0.950
-493G/T SNP (GG × GT) × BMI (≥ 25 kg/m^2^)	0.98	0.32‒3.00	0.969
-493G/T SNP (GG × TT) × BMI (≥ 25 kg/m^2^)	1.522	0.235‒9.85	0.660
-493G/T SNP (GG × GT) × HOMA-IR (≥ 3)	0.67	0.21‒2.18	0.508
-493G/T SNP (GG × TT) × HOMA-IR (≥ 3)	2.25	0.30‒17.17	0.434
-493G/T SNP (GG × GT) × Hypertension	1.14	0.37‒3.57	0.818
-493G/T SNP (GG × TT) × Hypertension	3.65	0.48‒27.70	0.211
-493G/T SNP (GG × GT) × Diabetes mellitus	0.72	0.15‒3.44	0.684
-493G/T SNP (GG × TT) × Diabetes mellitus	1.40	0.09‒21.20	0.807
-493G/T SNP (GG × GT) × HCV genotype 3	11.72	2.15‒63.82	**0.004**
-493G/T SNP (GG × TT) × HCV genotype 3	4.72	0.36‒62.03	0.237
-493G/T SNP (GG × GT) × ALT (≥41 U/L)	1.08	0.36‒3.22	0.892
-493G/T SNP (GG × TT) × ALT (≥41 U/L)	3.96	0.55‒28.38	0.171
-493G/T SNP (GG × GT) × AST (≥37 U/L)	1.48	0.49‒4.46	0.237
-493G/T SNP (GG × TT) × AST (≥37 U/L)	4.92	0.65‒37.07	0.122
-493G/T SNP (GG × GT) × GGT (≥61 U/L)	1.56	0.52‒4.68	0.424
-493G/T SNP (GG × TT) × GGT (≥61 U/L)	8.38	0.74‒4.52	0.085
-493G/T SNP (GG × GT) × Total cholesterol (≥ 200 mg/dL)	0.38	0.10‒1.38	0.139
-493G/T SNP (GG × TT) × Total cholesterol (≥ 200 mg/dL)	0.29	0.04‒2.30	0.242
-493G/T SNP (GG × GT) × LDL (≥ 130 mg/dL)	0.41	0.09‒1.80	0.237
-493G/T SNP (GG × TT) × LDL (≥ 130 mg/dL)	0.95	0.06‒15.09	0.970
-493G/T SNP (GG × GT) × HDL (≤ 60 mg/dL)	0.54	0.17‒1.74	0.300
-493G/T SNP (GG × TT) × HDL (≤ 60 mg/dL)	1.68	0.26‒10.97	0.586
-493G/T SNP (GG × GT) × VLDL (≥ 40 mg/dL)	3.00	0.29‒30.86	0.356
-493G/T SNP (GG × TT) × VLDL (≥ 40 mg/dL)	1.71	0.09‒33.91	0.732
-493G/T SNP (GG × GT) × Triglyceride (≥ 200 mg/dL)	2.20	0.20‒24.25	0.518
-493G/T SNP (GG × TT) × Triglyceride (≥ 200 mg/dL)	1.71	0.09‒33.91	0.723
-493G/T SNP (GG × GT) × Fibrosis (F3‒F4)	2.46	0.58‒10.44	0.221
-493G/T SNP (GG × TT) × Fibrosis (F3‒F4)	1.56	0.12‒20.47	0.737
-493G/T SNP (GG × GT) × Inflammatory activity (A2‒A3)	2.45	0.69‒8.68	0.165
-493G/T SNP (GG × TT) × Inflammatory activity (A2‒A3)	4.92	0.55‒43.78	0.153
-493G/T SNP (GG × GT) × Siderosis	1.27	0.13‒12.57	0.836
-493G/T SNP (GG × TT) × Siderosis	&		>0.999
**Recessive model**			
-493G/T SNP GG/GT	1.66	0.70‒3.93	0.246
-493G/T SNP × Sex (female)	0.77	0.14‒4.44	0.773
-493G/T SNP × Age (≥ 50 years)	1.06	0.11‒9.92	0.963
-493G/T SNP × BMI (≥ 25 kg/m^2^)	1.53	0.26‒9.05	0.639
-493G/T SNP × HOMA-IR (≥3)	2.74	0.39‒19.16	0.311
-493G/T SNP × Hypertension	3.43	0.49‒23.91	0.213
-493G/T SNP × Diabetes mellitus	1.72	0.14‒21.90	0.675
-493G/T SNP × HCV genotype 3	1.40	0.12‒16.33	0.787
-493G/T SNP × ALT (≥ 41 U/L)	3.82	0.57‒25.47	0.167
-493G/T SNP × AST (≥ 37 U/L)	4.10	0.58‒28.76	0.156
-493G/T SNP × GGT (≥ 61 U/L)	6.76	0.64‒71.99	0.113
-493G/T SNP × Total cholesterol (≥ 200 mg/dL)	0.45	0.06‒3.27	0.432
-493G/T SNP × LDL (≤ 130 mg/dL)	1.52	0.11‒21.62	0.757
-493G/T SNP × HDL (≤ 60 mg/dL)	2.28	0.38‒13.58	0.366
-493G/T SNP × VLDL (≥ 40 mg/dL)	1.04	0.06‒16.94	0.978
-493G/T SNP × Triglyceride (≥ 200 mg/dL)	1.23	0.07‒20.29	0.887
-493G/T SNP × Fibrosis (F3‒F4)	0.98	0.08‒11.62	0.987
-493G/T SNP × Inflammatory activity (A2‒A3)	3.34	0.40‒27.83	0.265
-493G/T SNP × Siderosis	&		>0.999

a Bivariate test. A significance level of *p* < 0.20 is marked in bold font. & Indicates that the estimation was not possible. ALT, alanine aminotransferase; AST, aspartate aminotransferase; BMI, body mass index; CI, confidence interval; GGT, gamma glutamyl transpeptidase; HCV, hepatitis C virus; HDL, high-density lipoprotein; HOMA-IR, homeostasis model assessment of insulin resistance; LDL, low-density lipoprotein; OR, odds ratio; SNP, single nucleotide polymorphism; VLDL, very low-density lipoprotein.

**Table 3:** Result of bivariate tests for interactions of the I128T SNP in the *MTTP* gene in different genetic models with characteristics of interest that influence the presence of hepatic steatosis in patients with chronic hepatitis C.

Parameter	OR	95% CI	p^a^
**Dominant model**			
II128T SNP IT/TT	1.07	0.64‒1.78	0.800
II128T SNP × Sex (female)	0.79	0.28‒2.23	0.657
II128T SNP × Age (≥ 50 years)	1.51	0.46‒4.96	0.495
II128T SNP × BMI (≥ 25 kg/m^2^)	0.88	0.31‒2.54	0.818
II128T SNP × HOMA-IR (≥ 3)	0.93	0.31‒2.82	0.897
II128T SNP × Hypertension	1.42	0.48‒4.20	0.532
II128T SNP × Diabetes mellitus	1.12	0.26‒4.73	0.881
II128T SNP × HCV genotype 3	7.90	1.67‒37.29	**0.009**
II128T SNP × ALT (≥ 41 U/L)	0.97	0.34‒2.76	0.960
II128T SNP × AST (≥ 37 U/L)	1.20	0.42‒3.43	0.732
II128T SNP × GGT (≥ 61 U/L)	2.39	0.83‒6.82	0.105
II128T SNP × Total cholesterol (≥ 200 mg/dL)	0.44	0.13‒1.48	0.184
II128T SNP × HDL (≤ 60 mg/dL)	0.74	0.24‒2.22	0.588
II128T SNP × LDL (≥ 130 mg/dL)	0.44	0.11‒1.78	0.246
II128T SNP × VLDL (≥ 40 mg/dL)	2.67	0.32‒21.94	0.362
II128T SNP × Triglyceride (≥ 200 mg/dL)	2.19	0.26‒18.52	0.474
II128T SNP × Fibrosis (F3‒F4)	1.90	0.48‒7.51	0.363
II128T SNP × Inflammatory activity (A2‒A3)	1.52	0.46‒4.98	0.488
II128T SNP × Siderosis	0.92	0.10‒8.73	0.940
**Codominant model**			
II128T SNP II × IT	0.90	0.53‒1.54	0.699
II128T SNP II × TT	2.45	0.90‒6.69	0.081
II128T SNP (II × IT) × Sex (female)	0.84	0.28‒2.49	0.747
II128T SNP (II × TT) × Sex (female)	0.44	0.05‒3.61	0.443
II128T SNP (II × IT) × Age (≥ 50 years)	1.42	0.40‒5.01	0.592
II128T SNP (II × TT) × Age (≥ 50 years)	2.11	0.20‒22.74	0.538
II128T SNP (II × IT) × BMI (≥ 25 kg/m^2^)	0.94	0.31‒2.86	0.914
II128T SNP (II × TT) × BMI (≥ 25 kg/m^2^)	0.832	0.106‒6.50	0.861
II128T SNP (II × IT) × HOMA‒IR (≥3)	0.71	0.22‒2.29	0.569
II128T SNP (II × TT) × HOMA‒IR (≥3)	3.35	0.28‒40.66	0.342
II128T SNP (II × IT) × Hypertension	1.08	0.35‒3.37	0.894
II128T SNP (II × TT) × Hypertension	6.74	0.55‒82.56	0.136
II128T SNP (II × IT) × Diabetes mellitus	1.12	0.25‒5.07	0.884
II128T SNP (II × TT) × Diabetes mellitus	1.09	0.07‒16.48	0.949
II128T SNP (II × IT) × HCV genotype 3	7.45	1.53‒36.23	**0.013**
II128T SNP (II × TT) × HCV genotype 3	&		0.999
II128T SNP (II × IT) × ALT (≥ 41 U/L)	0.81	0.27‒2.43	0.712
II128T SNP (II × TT) × ALT (≥ 41 U/L)	1.82	0.22‒14.77	0.576
II128T SNP (II × IT) × AST (≥ 37 U/L)	1.08	0.36‒3.26	0.887
II128T SNP (II × TT) × AST (≥ 37 U/L)	1.35	0.17‒10.95	0.780
II128T SNP (II × IT) × GGT (≥ 61 U/L)	1.97	0.66‒5.89	0.227
II128T SNP (II × TT) × GGT (≥ 61U/L)	&		0.999
II128T SNP (II × IT) × Total cholesterol (≥ 200 mg/dL)	0.45	0.12‒1.63	0.222
II128T SNP (II × TT) × Total cholesterol (≥ 200 mg/dL)	0.33	0.03‒3.22	0.342
II128T SNP (II × IT) × LDL (≤ 130 mg/dL)	0.42	0.10‒1.85	0.251
II128T SNP (II × TT) × LDL (≤ 130 mg/dL)	0.61	0.04‒9.96	0.727
II128T SNP (II × IT) × HDL (≤ 60 mg/dL)	0.61	0.19‒1.97	0.407
II128T SNP (II × TT) × HDL (≤ 60 mg/dL)	2.27	0.29‒17.93	0.438
II128T SNP (II × IT) × VLDL (≥ 40 mg/dL)	3.18	0.31‒32.67	0.331
II128T SNP (II × TT) × VLDL (≥ 40 mg/dL)	1.06	0.05‒22.00	0.969
II128T SNP (II × IT) × Triglyceride (≥ 200 mg/dL)	2.33	0.21‒25.63	0.489
II128T SNP (II × TT) × Triglyceride (≥ 200 mg/dL)	1.06	0.05‒22.00	0.969
II128T SNP (II × IT) × Fibrosis (F3‒F4)	2.27	0.54‒9.57	0.265
II128T SNP (II × TT) × Fibrosis (F3‒F4)	0.88	0.06‒12.14	0.923
II128T SNP (II × IT) × Inflammatory activity (A2‒A3)	1.60	0.44‒5.84	0.478
II128T SNP (II × TT) × Inflammatory activity (A2‒A3)	2.24	0.23‒21.35	0.484
II128T SNP (II × IT) × Siderosis	0.81	0.08‒8.36	0.862
II128T SNP (II × TT) × Siderosis	&		>0.999
**Recessive model**			
II128T SNP II/IT	2.57	0.97‒6.82	0.058
II128T SNP × Sex (female)	0.48	0.06‒3.69	0.476
II128T SNP × Age (≥ 50 years)	1.93	0.19‒19.87	0.579
II128T SNP × BMI (≥ 25 kg/m^2^)	0.85	0.12‒6.26	0.877
II128T SNP × HOMA‒IR (≥ 3)	3.94	0.35‒44.92	0.269
II128T SNP × Hypertension	6.62	0.58‒75.90	0.129
II128T SNP × Diabetes mellitus	1.04	0.08‒13.99	0.976
II128T SNP × HCV genotype 3	&		0.999
II128T SNP × ALT (≥ 41 U/L)	1.99	0.26‒15.22	0.507
II128T SNP × AST (≥ 37 U/L)	1.30	0.17‒9.96	0.800
II128T SNP × GGT (≥ 61 U/L)	&		0.999
II128T SNP × Total cholesterol (≥ 200 mg/dL)	0.46	0.05‒4.17	0.490
II128T SNP × LDL (≤ 130 mg/dL)	0.92	0.06‒13.76	0.950
II128T SNP × HDL (≤ 60 mg/dL)	2.83	0.38‒20.96	0.309
II128T SNP × VLDL (≥ 40 mg/dL)	0.63	0.04‒10.81	0.751
II128T SNP × Triglyceride ≥ 200 (mg/dL)	0.74	0.04‒12.94	0.839
II128T SNP × Fibrosis (F3‒F4)	0.59	0.05‒7.44	0.685
II128T SNP × Inflammatory activity (A2‒A3)	1.91	0.21‒17.24	0.564
II128T SNP × Siderosis	&		>0.999

a Bivariate test. A significance level of *p* < 0.20 is marked in bold font. & Indicates that the estimation was not possible. ALT, alanine aminotransferase; AST, aspartate aminotransferase; BMI, body mass index; CI, confidence interval; GGT, gamma glutamyl transpeptidase; HCV, hepatitis C virus; HDL, high-density lipoprotein; HOMA-IR, homeostasis model assessment of insulin resistance; LDL, low-density lipoprotein; OR, odds ratio; SNP, single nucleotide polymorphism; VLDL, very low-density lipoprotein.

**Table 4:** Result of bivariate tests for interactions of the Q95H SNP in the *MTTP* gene with characteristics of interest that influence the presence of hepatic steatosis in patients with chronic hepatitis C.

Parameter	OR	95% CI	p^a^
**Dominant model**^b^			
Q95H SNP QH/HH	2.08	1.01‒4.26	0.046
Q95H SNP × Sex (female)	1.41	0.32‒6.13	0.649
Q95H SNP × Age (≥50 years)	0.92	0.16‒5.47	0.929
Q95H SNP × BMI (≥25 kg/m^2^)	0.14	0.02‒0.80	**0.027**
Q95H SNP × HOMA‒IR (≥3)	0.95	0.20‒4.45	0.943
Q95H SNP × Hypertension	0.87	0.20‒3.73	0.852
Q95H SNP × Diabetes mellitus	3.72	0.35‒39.69	0.277
Q95H SNP × HCV genotype 3	0.36	0.06‒2.11	0.259
Q95H SNP × ALT (≥41 U/L)	0.88	0.21‒3.79	0.865
Q95H SNP × AST (≥37 U/L)	1.21	0.28‒5.33	0.801
Q95H SNP × GGT (≥61 U/L)	0.59	0.14‒2.52	0.479
Q95H SNP × Total cholesterol (≥200 mg/dL)	1.76	0.27‒11.45	0.557
Q95H SNP × LDL (≥130 mg/dL)	1.29	0.18‒9.49	0.802
Q95H SNP × HDL (≤60 mg/dL)	0.50	0.08‒3.06	0.450
Q95H SNP × VLDL (≥40 mg/dL)	0.57	0.06‒5.55	0.626
Q95H SNP × Triglyceride (≥200 mg/dL)	0.69	0.07‒6.92	0.750
Q95H SNP × Fibrosis (F3‒F4)	3.15	0.31‒31.64	0.329
Q95H SNP × Inflammatory activity (A2‒A3)	0.41	0.08‒1.98	0.264
Q95H SNP × Siderosis	0.19	0.01‒4.32	0.300

a Bivariate test. A significance level of *p* < 0.20 is marked in bold font.

b Because only one patient presented the HH genotype, it was not possible to perform the analyses in other genetic models. ALT, Alanine Aminotransferase; AST, Aspartate Aminotransferase; BMI, Body Mass Index; CI, Confidence Interval; GGT, Gamma Glutamyl Transpeptidase; HCV, Hepatitis C Virus; HDL, High-Density Lipoprotein; HOMA-IR, Homeostasis Model Assessment of Insulin Resistance; LDL, Low-Density Lipoprotein; OR, Odds Ratio; SNP, Single Nucleotide Polymorphism; VLDL, Very Low-Density Lipoprotein.

**Table 5:** Result of bivariate tests for interactions of the Q244E SNP in the *MTTP* gene with characteristics of interest that influence the presence of hepatic steatosis in patients with chronic hepatitis C.

Parameter	OR	95% CI	p^a^
Q244E SNP (QE × EE)^b^	1.33	0.59‒3.01	0.487
Q244E SNP × Sex (female)	3.52	0.58‒21.56	0.174
Q244E SNP × Age (≥50 years)	3.39	0.46‒24.87	0.229
Q244E SNP × BMI (≥25 kg/m^2^)	1.06	0.21‒5.45	0.947
Q244E SNP × HOMA‒IR (≥3)	0.81	0.15‒4.44	0.810
Q244E SNP × Hypertension	0.90	0.17‒4.83	0.905
Q244E SNP × Diabetes mellitus	0.92	0.11‒7.65	0.935
Q244E SNP × HCV genotype 3	&		
Q244E SNP × ALT (≥41 U/L)	1.08	0.19‒6.24	0.936
Q244E SNP × AST (≥37 U/L)	1.10	0.20‒5.92	0.912
Q244E SNP × GGT (≥61 U/L)	0.41	0.07‒2.28	0.309
Q244E SNP × Total cholesterol ≥200 mg/dL)	7.88	0.73‒85.12	0.089
Q244E SNP × LDL (≥130 mg/dL)	3.39	0.27‒42.14	0.342
Q244E SNP × HDL (≤60 mg/dL)	0.63	0.12‒3.40	0.595
Q244E SNP × VLDL (≥40 mg/dL)	&		
Q244E SNP × Triglyceride (≥200 mg/dL)	&		
Q244E SNP × Fibrosis (F3‒F4)	0.74	0.10‒5.44	0.763
Q244E SNP × Inflammatory activity (A2‒A3)	0.73	0.13‒4.23	0.726
Q244E SNP × Siderosis	&		

a Bivariate test. Significance level of *p* < 0.20.

b No patients presented the QQ genotype. & Indicates that the estimation was not possible. ALT, Alanine Aminotransferase; AST, Aspartate Aminotransferase; BMI, Body Mass Index; CI, Confidence Interval; GGT, Gamma Glutamyl Transpeptidase; HCV, Hepatitis C Virus; HDL, High-Density Lipoprotein; HOMA-IR, Homeostasis Model Assessment of Insulin Resistance; LDL, Low-Density Lipoprotein; OR, Odds Ratio; SNP, Single Nucleotide Polymorphism; VLDL, Very Low-Density Lipoprotein.

**Table 6:** Result of multivariate tests for interactions of the ‒493G/T SNP in the *MTTP* gene (in different genetic models) with characteristics of interest that influence the presence of hepatic steatosis in patients with chronic hepatitis C.

Parameter	OR	95% CI	p^a^
**Dominant model**			
Sex (female)	1.94	1.07‒3.50	0.028
HOMA-IR (≥3)	1.87	1.01‒3.47	0.048
HCV genotype 3	0.73	0.22‒2.40	0.601
Inflammatory activity (A2‒A3)	4.75	2.51‒8.97	< 0.001
-493G/T SNP (GT/TT)	0.66	0.35‒1.24	0.193
-493G/T SNP (GT/TT) × HCV genotype 3	11.51	2.08‒63.59	**0.005**
**Codominant model**			
Sex (female)	1.92	1.06‒3.49	0.031
HOMA-IR (≥3)	1.88	1.00‒3.50	0.048
HCV genotype 3	0.72	0.22‒2.40	0.597
Inflammatory activity (A2‒A3)	4.94	2.60‒9.40	< 0.001
-493G/T SNP (GG)			
GT	0.56	0.29‒1.10	0.091
TT	1.25	0.42‒3.75	0.690
-493G/T SNP (GG) × HCV genotype 3			
-493G/T SNP (GT) × HCV genotype 3	15.69	2.46‒99.85	**0.004**
-493G/T SNP (TT) × HCV genotype 3	3.64	0.23‒56.51	0.356
**Recessive model**			
Sex (female)	2.08	1.16‒3.73	0.014
HOMA-IR (≥3)	1.71	0.93‒3.15	0.084
HCV genotype 3	2.78	1.27‒6.11	0.011
Inflammatory activity (A2‒A3)	4.52	2.43‒8.39	< 0.001
-493G/T SNP (GG/GT)	1.62	0.62‒4.22	0.327

a Multiple logistic regression. When interaction is placed, only the interaction should be interpreted. The significance level of *p* < 0.05 is marked in bold font. CI, Confidence Interval; HCV, Hepatitis C Virus; HOMA-IR, Homeostasis Model Assessment of Insulin Resistance; OR, Odds Ratio; SNP, Single Nucleotide Polymorphism.

**Table 7:** Result of multivariate tests for interactions of the I128T SNP in the *MTTP* gene (in different genetic models) with characteristics of interest that influence the presence of hepatic steatosis in patients with chronic hepatitis C.

Parameter	OR	95% CI	p^a^
**Dominant model**			
Sex (female)	1.99	1.11‒3.59	0.022
HOMA-IR (≥3)	1.84	0.99‒341	0.053
HCV genotype 3	0.91	0.29‒2.88	0.877
Inflammatory activity (A2‒A3)	4.67	2.48‒8.79	< 0.001
I128T SNP (IT/TT)	0.64	0.34‒1.20	0.166
I128T SNP (IT/TT) × HCV genotype 3	8.51	1.59‒45.54	**0.012**
**Codominant model**			
Sex (female)	2.01	1.10‒3.64	0.022
HOMA-IR (≥3)	1.76	0.94‒3.28	0.077
HCV genotype 3	0.92	0.29‒2.91	0.885
Inflammatory activity (A2‒A3)	4.97	2.60‒9.48	< 0.001
I128T SNP (II)			
IT	0.51	0.26‒1.01	0.052
TT	1.66	0.52‒5.33	0.392
I128T SNP (II) × HCV genotype 3			
I128T SNP (IT) × HCV genotype 3	8.40	1.51‒46.91	**0.015**
I128T SNP (TT) × HCV genotype 3	&		0.999
**Recessive model**			
Sex (female)	2.08	1.15‒3.74	0.015
HOMA-IR (≥3)	1.68	0.91‒3.10	0.098
HCV genotype 3	2.82	1.28‒6.22	0.010
Inflammatory activity (A2‒A3)	4.63	2.48‒8.66	< 0.001
I128T SNP (II/IT)	2.67	0.92‒7.81	0.072

a Multiple logistic regression. When interaction is placed, only the interaction should be interpreted. The significance level of *p* < 0.05 is marked in bold font. & Indicates that the estimation was not possible. CI, Confidence Interval; HCV, Hepatitis C Virus; HOMA-IR, Homeostasis Model Assessment of Insulin Resistance; OR, Odds Ratio; SNP, Single Nucleotide Polymorphism.

**Table 8:** Result of multivariate tests for interactions of the Q95H SNP in the *MTTP* gene (in different genetic models) with characteristics of interest that influence the presence of hepatic steatosis in patients with chronic hepatitis C.

Parameter	OR	95% CI	p^a^
**Dominant model**^b^			
Sex (female)	2.23	1.22‒4.09	0.009
HOMA-IR (≥3)	1.60	0.85‒3.01	0.145
HCV genotype 3	2.52	1.13‒5.62	0.024
Inflammatory activity (A2‒A3)	5.08	2.65‒9.75	< 0.001
BMI (≥25)	1.33	0.69‒2.55	0.397
Q95H SNP (QH/HH)	7.89	1.44‒43.12	0.017
Q95H SNP (QH/HH) × BMI (≥25)	0.15	0.02‒1.03	0.053

a Multiple logistic regression. When interaction is placed, only the interaction should be interpreted. Significance level of *p* < 0.05.

b Because only one patient presented the HH genotype, it was not possible to perform the analyses in other genetic models. BMI, Body Mass Index; CI, Confidence Interval; HCV, Hepatitis C Virus; HOMA-IR, Homeostasis Model Assessment of Insulin Resistance; OR, Odds Ratio; SNP, Single Nucleotide Polymorphism.

**Table 9:** Result of multivariate tests for interactions of the Q244E SNP in the *MTTP* gene (in different genetic models) with characteristics of interest that influence the presence of hepatic steatosis in patients with chronic hepatitis C.

Parameter	OR	95% CI	p^a^
Sex (female)	2.05	1.14‒3.67	0.016
HOMA-IR (≥3)	1.73	0.94‒3.18	0.079
HCV genotype	2.82	1.29‒6.17	0.009
Inflammatory activity (A2‒A3)	4.52	2.44‒8.39	<0.001
Q244E SNP (QE × EE)^b^	1.29	0.50‒3.31	0.597

a Multiple logistic regression. When interaction is placed, only the interaction should be interpreted. Significance level of *p* < 0.05.

b No patients presented the QQ genotype. CI, Confidence Interval; HCV, Hepatitis C Virus; HOMA-IR, Homeostasis Model Assessment of Insulin Resistance; OR, Odds Ratio; SNP, Single Nucleotide Polymorphism.

In the final multiple logistic regression model used to determine which characteristics combined with each SNP to influence the presence of hepatic steatosis in patients with chronic hepatitis C, it was observed that in the dominant genetic model (GG × GT/TT), the GT/TT genotype of the -493G/T SNP in the *MTTP* gene combined with HCV genotype 3 infection presented an 11.51-fold higher risk of hepatic steatosis than that observed in carriers of the GG genotype without HCV genotype 3 infections (95% CI 2.08‒63.59, *p* = 0.005). Similar results were observed in the codominant model (GG × GT × TT) of the -493G/T SNP, in which carriers of the GT genotype combined with HCV genotype 3 infection presented a 15.69-fold higher risk of hepatic steatosis than that observed in carriers of the GG genotype without HCV genotype 3 infections (95% CI 2.46‒99.85, *p* = 0.004) (Table 6).

In the dominant genetic model (II × IT/TT), the IT/TT genotype of the I128T SNP in the *MTTP* gene combined with HCV genotype 3 infection presented an 8.51-fold higher risk of hepatic steatosis than that observed in carriers of the II genotype without HCV genotype 3 infections (95% CI 1.59‒45.54, *p* = 0.012). In the codominant model (II × IT × TT), carriers of the IT genotype of the I128T SNP combined with HCV genotype 3 infection presented an 8.40-fold higher risk of hepatic steatosis than that observed in carriers of the II genotype without HCV genotype 3 infections (95% CI 1.51‒46.91, *p* = 0.015) (Table 7). The Q95H and Q244E SNPs in the *MTTP* gene did not influence the presence of hepatic steatosis in this group of patients when combined with other variables (Tables 8 and 9).

## Discussion

Hepatitis C is a frequent liver disease found worldwide. Even in the era of DAAs, the determination of genetic markers is important to identify individuals at higher risk of severe disease and to guide therapeutic decisions prior to therapy in patients with chronic hepatitis C.^22^ In the present study, the authors analyzed the effect of four candidate SNPs in the *MTTP* gene combined with host and viral characteristics on hepatic steatosis in a group of chronic hepatitis C patients. Multiple logistic regression analysis for interaction showed an 11.51-fold higher risk of steatosis in patients with the GT/TT genotype of the -493G/T SNP and HCV genotype 3 infections. The same results were observed when another genetic model, the codominant model, was analyzed, in which carriers of the GT genotype combined with HCV genotype 3 infection presented a 15.69-fold higher risk of steatosis. The present study also investigated the I128T SNP of the *MTTP* gene, and the risk of steatosis was 8.51-fold higher in patients with the IT/TT genotype of the I128T SNP when combined with HCV genotype 3 infections. In another genetic model, the codominant model, carriers of the IT genotype combined with HCV genotype 3 infection presented an 8.40-fold higher risk of steatosis.

Several studies have explored the association between the -493G/T and I128T SNPs of the *MTTP* gene and NAFLD. However, the effect of these polymorphisms on NAFLD remains uncertain due to the inconsistent results of different studies. A recent meta‑analysis evaluated these SNPs under different genetic models on NASH and NAFLD, demonstrating that the -493G/T SNP is associated with NASH susceptibility (determined by liver biopsy).^23^

The role of the -493G/T SNP in the *MTTP* gene associated with hepatitis C has been thoroughly studied, but different results have been reported.^24^, ^25^, ^26^, ^27^, ^28^ Akgöllü and Akkız^29^ evaluated the relationship of the -493G/T SNP with hepatic steatosis in a Turkish population with HCV genotype 1 infection. Despite finding a statistically significant association between levels of triglycerides, total cholesterol, LDL, and VLDL and the number of patients with steatosis, these researchers reported that the studied polymorphism is not associated with the presence of steatosis in individuals who exhibited HCV genotype 1 infection.^29^ A previous Swiss cohort study, including 443 patients infected with HCV genotype non 3 and 183 patients infected with HCV genotype 3, reported that the -493G/T SNP is associated with the presence of steatosis in multivariate analysis only in patients with HCV genotype non 3.^27^ However, in another study evaluating 102 treatment-naïve patients for hepatitis C it was reported that patients infected with HCV genotype 3 and with the T (mutated) allele of the -493G/T SNP are associated with hepatic steatosis, which corroborates the present findings. Furthermore, these patients also present a higher grade of inflammation in the liver and more liver fibrosis as well as higher HCV-RNA serum levels than those observed in carriers of the wild-type allele (G).^25^

Regarding the I128T SNP in the *MTTP* gene, previous multivariate analyses conducted in a Han Chinese population have indicated that this SNP is not associated with NAFLD.^30^ However, this polymorphism has been associated with the presence of central obesity, elevated liver enzymes, and alcoholic fatty liver disease in Koreans.^31^ Hashemi et al.^32^ investigated the association between the I128T and Q95H SNPs in the *MTTP* gene in a sample of Iranian patients with NAFLD, and they observed that the IT genotype and the IT+TT genotype of the I128T SNP increase susceptibility to NAFLD. In addition, it has been reported that there is no association between the Q95H SNP and NAFLD.^32^

Hepatic steatosis is a frequent histological feature among patients with chronic hepatitis C, and it significantly affects disease progression.^33^ In the present study, hepatic steatosis was observed in 53% of the patients, which was similar to the mean prevalence of 55% previously described by Asselah et al.^34^ Host and viral factors are involved in the development and severity of HCV-associated steatosis. Recently, several SNPs have been reported to be associated with alterations in hepatic fibrosis and steatosis even after DAA therapy for HCV infection, indicating that they may have prognostic value for the assessment of post-SVR evolution.^35^^,^^36^

The present study had several limitations. The sample size was limited, and the study was conducted in a single center. To validate the present findings, a study including a larger number of samples should be performed in the future. However, the present results may aid in the establishment of genetic markers to predict hepatic steatosis.

## Conclusions

In summary, the present study highlighted the significant role of *MTTP* SNPs in the pathogenesis of hepatic steatosis in hepatitis C. The association analysis of the group of patients with chronic hepatitis C provides useful information on the effect of HCV genotype 3 infections combined with the -493G/T and I128T SNPs in the *MTTP* gene on hepatic steatosis. These findings may have prognostic importance in patients with chronic hepatitis C and help guide decision-making for appropriate follow-up and treatment for those at increased risk of liver disease progression.

## Conflicts of interest

The authors declare no conflicts of interest.

## Appendix Supplementary materials

Image, application 1

## References

[1] Sharma A, Nagalli S. (2022). StatPearls.

[2] World Health Organization (2021). https://www.who.int/news-room/fact-sheets/detail/hepatitis-c.

[3] World Health Organization (2021).

[4] Chaudhari R, Fouda S, Sainu A, Pappachan JM. (2021). Metabolic complications of hepatitis C virus infection. World J Gastroenterol.

[5] Rout G, Nayak B, Patel AH, Gunjan D, Singh V, Kedia S (2019). Therapy with oral directly acting agents in hepatitis c infection is associated with reduction in fibrosis and increase in hepatic steatosis on transient elastography. J Clin Exp Hepatol.

[6] Kawagishi N, Suda G, Nakamura A, Kimura M, Maehara O, Suzuki K (2018). Liver steatosis and dyslipidemia after HCV eradication by direct acting antiviral agents are synergistic risks of atherosclerosis. PLoS One.

[7] Soliman H, Ziada D, Hamisa M, Badawi R, Hawash N, Salama M (2022). The effect of HCV eradication after direct-acting antiviral agents on hepatic steatosis: a prospective observational study. Endocr Metab Immune Disord Drug Targets.

[8] Pontual DM, Nabuco LC, Luiz RR, Cardoso AC, Perez RM, Villela-Nogueira CA. (2021). Diabetes influences liver stiffness in chronic hepatitis C patients with and without virological cure: a longitudinal study. Clinics.

[9] Valenti L, Rumi M, Galmozzi E, Aghemo A, Del Menico D, De Nicola S (2011). Patatin-like phospholipase domain-containing 3 I148M polymorphism, steatosis, and liver damage in chronic hepatitis C. Hepatology.

[10] Adinolfi LE, Restivo L, Marrone A. (2013). The predictive value of steatosis in hepatitis C virus infection. Expert Rev Gastroenterol Hepatol.

[11] Magri MC, Prata TVG, Manchiero C, Dantas BP, Mazza CC, Tengan FM. (2017). Genetic variation in the microsomal triglyceride transfer protein (-493G/T) is associated with hepatic steatosis in patients infected with hepatitis C virus. BMC Infect Dis.

[12] Manchiero C, Nunes AKS, Magri MC, Dantas BP, Mazza CC, Barone AA (2017). The rs738409 polymorphism of the PNPLA3 gene is associated with hepatic steatosis and fibrosis in Brazilian patients with chronic hepatitis C. BMC Infect Dis.

[13] Stevenson HL, Utay NS. (2016). Hepatic steatosis in HCV-infected persons in the direct-acting antiviral era. Trop Dis Travel Med Vaccines.

[14] Prata TVG, Silva DSRD, Manchiero C, Dantas BP, Mazza CC, Nunes AKS (2019). MTTP polymorphisms and hepatic steatosis in individuals chronically infected with hepatitis C virus. Arch Virol.

[15] Kirkwood BR, Sterne JAC. (2006).

[16] Mirandola S, Osterreicher CH, Marcolongo M, Datz C, Aigner E, Schlabrakowski A (2009). Microsomal triglyceride transfer protein polymorphism (-493G/T) is associated with hepatic steatosis in patients with chronic hepatitis C. Liver Int.

[17] Kleiner DE, Brunt EM, Van Natta M, Behling C, Contos MJ, Cummings OW (2005). Nonalcoholic Steatohepatitis Clinical Research Network. Design and validation of a histological scoring system for nonalcoholic fatty liver disease. Hepatology.

[18] The French METAVIR Cooperative Study Group (1994). Intraobserver and interobserver variations in liver biopsy interpretation in patients with chronic hepatitis C. Hepatology.

[19] Karpe F, Lundahl B, Ehrenborg E, Eriksson P, Hamsten A. (1998). A common functional polymorphism in the promoter region of the microsomal triglyceride transfer protein gene influences plasma LDL levels. Arterioscler Thromb Vasc Biol.

[20] Ledmyr H, Karpe F, Lundahl B, McKinnon M, Skoglund-Andersson C, Ehrenborg E. (2002). Variants of the microsomal triglyceride transfer protein gene are associated with plasma cholesterol levels and body mass index. J Lipid Res.

[21] Hosmer DW, Lemeshow S. (2000).

[22] Hemeda AA, Mohamed AA, Aziz RK, Abdel-Hakeem MS, Ali-Tammam M (2021). Impact of IL10, MTP, SOD2, and APOE gene polymorphisms on the severity of liver fibrosis induced by HCV genotype 4. Viruses.

[23] Tan J, Zhang J, Zhao Z, Zhang J, Dong M, Ma X (2020). The association between SNPs rs1800591 and rs3816873 of the MTTP gene and nonalcoholic fatty liver disease: a meta-analysis. Saudi J Gastroenterol.

[24] Petit JM, Masson D, Minello A, Duvillard L, Galland F, Verges B (2006). Lack of association between microsomal triglyceride transfer protein gene polymorphism and liver steatosis in HCV-infected patients. Mol Genet Metab.

[25] Zampino R, Ingrosso D, Durante-Mangoni E (2008). Microsomal triglyceride transfer protein (MTP) -493G/T gene polymorphism contributes to fat liver accumulation in HCV genotype 3 infected patients. J Viral Hepat.

[26] Mirandola S, Osterreicher CH, Marcolongo M, Datz C, Aigner E, Schlabrakowski A (2009). Microsomal triglyceride transfer protein polymorphism (-493G/T) is associated with hepatic steatosis in patients with chronic hepatitis C. Liver Int.

[27] Cai T, Dufour JF, Muellhaupt B, Gerlach T, Heim M, Moradpour D (2011). Viral genotype-specific role of PNPLA3, PPARG, MTTP, and IL28B in hepatitis C virus-associated steatosis. J Hepatol.

[28] Siqueira ER, Oliveira CP, Correa-Giannella ML (2012). MTP -493G/T gene polymorphism is associated with steatosis in hepatitis C-infected patients. Braz J Med Biol Res.

[29] Akgöllü E, Akkız H. (2019). Association between hepatic steatosis and MTP gene -493G/T polymorphism in the patients with HCV genotype 1 infection. Infect Genet Evol.

[30] Peng XE, Wu YL, Lu QQ, Hu ZJ, Lin X (2014). MTTP polymorphisms and susceptibility to non-alcoholic fatty liver disease in a Han Chinese population. Liver Int.

[31] Jun DW, Han JH, Jang EC, Kim SH, Kim SH, Jo YJ (2009). Polymorphisms of microsomal triglyceride transfer protein gene and phosphatidylethanolamine N-methyltransferase gene in alcoholic and nonalcoholic fatty liver disease in Koreans. Eur J Gastroenterol Hepatol.

[32] Hashemi M, Hoseini H, Yaghmaei P, Moazeni-Roodi A, Bahari A, Hashemzehi N (2011). Association of polymorphisms in glutamate-cysteine ligase catalytic subunit and microsomal triglyceride transfer protein genes with nonalcoholic fatty liver disease. DNA Cell Biol.

[33] Mirandola S, Bowman D, Hussain MH, Alberti A. (2010). Hepatic steatosis in hepatitis C is a storage disease due to HCV interaction with microsomal triglyceride transfer protein (MTP). Nutr Metab (Lond).

[34] Asselah T, Rubbia-Brandt L, Marcellin P, Negro F. (2006). Steatosis in chronic hepatitis C: why does it really matter?. Gut.

[35] Gavril OI, Arhire LI, Gavrilescu O, Dranga M, Barboi O, Gavril RS (2021). Role of PNPLA3 in the assessment and monitoring of hepatic steatosis and fibrosis in patients with chronic hepatitis C infection who achieved a sustained virologic response. Medicina.

[36] Matsumoto K, Miyaaki H, Fukushima M, Sasaki R, Haraguchi M, Miuma S (2022). The impact of single-nucleotide polymorphisms on liver stiffness and controlled attenuation parameter in patients treated with direct-acting antiviral drugs for hepatitis C infection. Biomed Rep.

