# Supplementary material for: Effect of MTTP -493G/T, I128T, Q95H and Q244E polymorphisms on hepatic steatosis in patients with chronic hepatitis
Source: Clinics (Sao Paulo). 2022 Aug 23;77:100094. doi: 10.1016/j.clinsp.2022.100094 (PMC9424342; doi:10.1016/j.clinsp.2022.100094)
Supplement: Supplementary file 1 [file mmc1.docx]

**Table S1** Result of bivariate tests of general characteristics in patients with chronic hepatitis C associated with presence of hepatic steatosis.

| **Characteristic** | **OR** | **95% CI** | **p^a^** |
| --- | --- | --- | --- |
| Sex (female) | 1.69 | 1.01‒2.84 | **0.047** |
| Age (≥ 50 years) | 1.95 | 1.10‒3.46 | **0.021** |
| BMI (≥ 25 kg/m^2^) | 1.12 | 0.66‒1.90 | 0.669 |
| HOMA-IR (≥ 3) | 1.85 | 1.07‒3.22 | **0.028** |
| Alcohol consumption (≥ 20 g/day) | 1.28 | 0.75‒2.18 | 0.361 |
| Hypertension (Yes) | 1.50 | 0.88‒2.56 | **0.140** |
| Diabetes mellitus (Yes) | 1.13 | 0.55‒2.31 | 0.735 |
| HCV genotype 3 (Yes) | 2.72 | 1.32‒5.61 | **0.005** |
| HCV viral load (≥ 850,000 IU/mL) | 0.90 | 0.53‒1.53 | 0.699 |
| ALT (≥ 41 U/L) | 1.63 | 0.97‒2.74 | **0.066** |
| AST (≥ 37 U/L) | 2.44 | 1.45‒4.13 | **0.001** |
| GGT (> 61 U/L) | 1.74 | 1.03‒2.92 | **0.038** |
| Total cholesterol (≥ 200 mg/dL) | 0.94 | 0.52‒1.71 | 0.839 |
| LDL (≥ 130 mg/dL) | 0.77 | 0.38‒1.53 | 0.450 |
| HDL (≤ 60 mg/dL) | 0.84 | 0.48‒1.45 | 0.524 |
| VLDL (≥ 40 mg/dL) | 1.15 | 0.42‒3.21 | 0.785 |
| Triglyceride (≥ 200 mg/dL) | 1.02 | 0.36‒2.90 | 0.977 |
| Hepatic fibrosis (F3‒F4) | 2.69 | 1.36‒5.33 | **0.004** |
| Hepatic inflammatory activity (A2‒A3) | 4.68 | 2.61‒8.41 | **<0.001** |
| Hepatic siderosis (Yes) | 1.84 | 0.61‒5.57 | 0.272 |

^a^ Bivariate test. The significance level of p < 0.20 is marked in bold font.

Adapted from, Prata et al.[14]

ALT, Alanine Aminotransferase; AST, Aspartate Aminotransferase; BMI, Body Mass Index; CI, Confidence Interval; GGT, Gamma Glutamyl Transpeptidase; HCV, Hepatitis C Virus; HDL, High-Density Lipoprotein; HOMA-IR, Homeostasis Model Assessment of Insulin Resistance; LDL, Low-Density Lipoprotein; OR, Odds Ratio; VLDL, Very Low-Density Lipoprotein.

**Table S2** Result of multivariate tests of general characteristics in patients with chronic hepatitis C associated with the presence of hepatic steatosis.

| **Characteristic** | **OR** | **95% CI** | **p****^a^** |
| --- | --- | --- | --- |
| Sex (female) | 2.28 | 1.21‒4.28 | **0.011** |
| Age (≥ 50 years) | 1.28 | 0.66‒2.51 | 0.464 |
| HOMA-IR (≥ 3) | 1.49 | 0.78‒2.88 | 0.230 |
| Hypertension (Yes) | 0.83 | 0.43‒1.60 | 0.574 |
| HCV genotype 3 | 2.74 | 1.24‒6.06 | **0.013** |
| ALT (≥ 41 U/L) | 0.89 | 0.37‒2.12 | 0.784 |
| AST (≥ 37 U/L) | 1.36 | 0.54‒3.43 | 0.513 |
| GGT (> 61 U/L) | 1.23 | 0.63‒2.40 | 0.543 |
| Hepatic fibrosis (F3‒F4) | 1.47 | 0.66‒3.30 | 0.349 |
| Hepatic Inflammatory activity (A2‒A3) | 3.61 | 1.86‒7.01 | **<0.001** |

^a^ Multiple logistic regression. The significance level of p<0.05 is marked in bold font.

Adapted from, Prata et al.[14]

ALT, Alanine Aminotransferase; AST, Aspartate Aminotransferase; CI, Confidence Interval; GGT, Gamma Glutamyl Transpeptidase; HCV, Hepatitis C Virus; HOMA-IR, Homeostasis Model Assessment of Insulin Resistance; OR, Odds Ratio.
